# *Actinomyces neuii*: a case report of a rare cause of acute infective endocarditis and literature review

**DOI:** 10.1186/s12879-019-4149-2

**Published:** 2019-06-10

**Authors:** Wei-Teng Yang, Matthew Grant

**Affiliations:** 1 Department of Internal Medicine, Yale New Haven Health Bridgeport Hospital, 267 Grant Street, Bridgeport, CT 06610 USA; 2 Department of Internal Medicine, Section of Infectious Diseases, Yale School of Medicine, PO Box 208022, New Haven, CT 06510 USA

**Keywords:** *Actinomyces neuii*, *Actinomyces*, Infective endocarditis

## Abstract

**Background:**

Infective endocarditis caused by *Actinomyces* spp. is extremely rare. However, cases by new species of *Actinomyces* have been increasingly reported due to advances in laboratory techniques, and many of these species do not cause classic presentations of actinomycosis. *Actinomyces neuii* is reported to have a tendency to cause endovascular infection. The course of infective endocarditis caused by *Actinomyces* spp. is usually indolent.

**Case presentation:**

A 61-year-old man with history of infective endocarditis, end stage renal disease, and monoclonal gammopathy was admitted for an abrupt fever, confusion, dysarthria, and facial droop after hemodialysis. Echocardiogram showed vegetations on both the aortic and mitral valves. Two sets of blood culture grew *A. neuii*. Brain MRI showed multiple bilateral cerebral infarcts consistent with septic emboli. The patient recovered after valvular surgery and prolonged intravenous and oral antibiotic therapy.

**Conclusions:**

This case illustrates an unusually acute presentation of *A. neuii* infective endocarditis. As with other Gram-positive bacilli, *Actinomyces* spp. isolates are often regarded as a result of contamination. One should keep it in mind as a cause of infective endocarditis in vulnerable patient populations.

## Background

*Actinomyces* spp. classically cause human actinomycosis, an indolent granulomatous infectious disease characterized by orocervicofacial, thoracic, abdominopelvic, or central nervous system abscess formation and draining sinuses [1]. Many novel *Actinomyces* species have been reported in recent decades with the advance in laboratory identification methods, and are associated with a wide range of infection at many body sites [2]. However, infective endocarditis by *Actinomyces* spp. is still extremely rare. We report a patient who presented with an acute *Actinomyces neuii* (*A. neuii*) aortic and mitral valve endocarditis complicated by aortic root abscess and septic cerebral emboli. He was treated successfully with surgery and prolonged antibiotics. We then present a review of published *Actinomyces* spp. endocarditis cases following a systematic literature search.

## Case presentation

### Clinical presentation and diagnostic findings

A 61 year-old man was admitted with a 103 ° F fever, confusion, weakness and slurred speech after hemodialysis. He had a history of viridans streptococcal mitral valve endocarditis, end stage renal disease on hemodialysis, atrial fibrillation not on anticoagulation due to GI bleeding, and monoclonal gammopathy of undetermined significance. He had a productive cough for a week without any identifiable sick contact. Physical examination was notable for an agitated edentulous man with a left central facial palsy, severe dysarthria, and a systolic murmur at the left lower sternal border. His lungs were clear to auscultation and there was no stigmata of endocarditis.

**Fig. 1:**
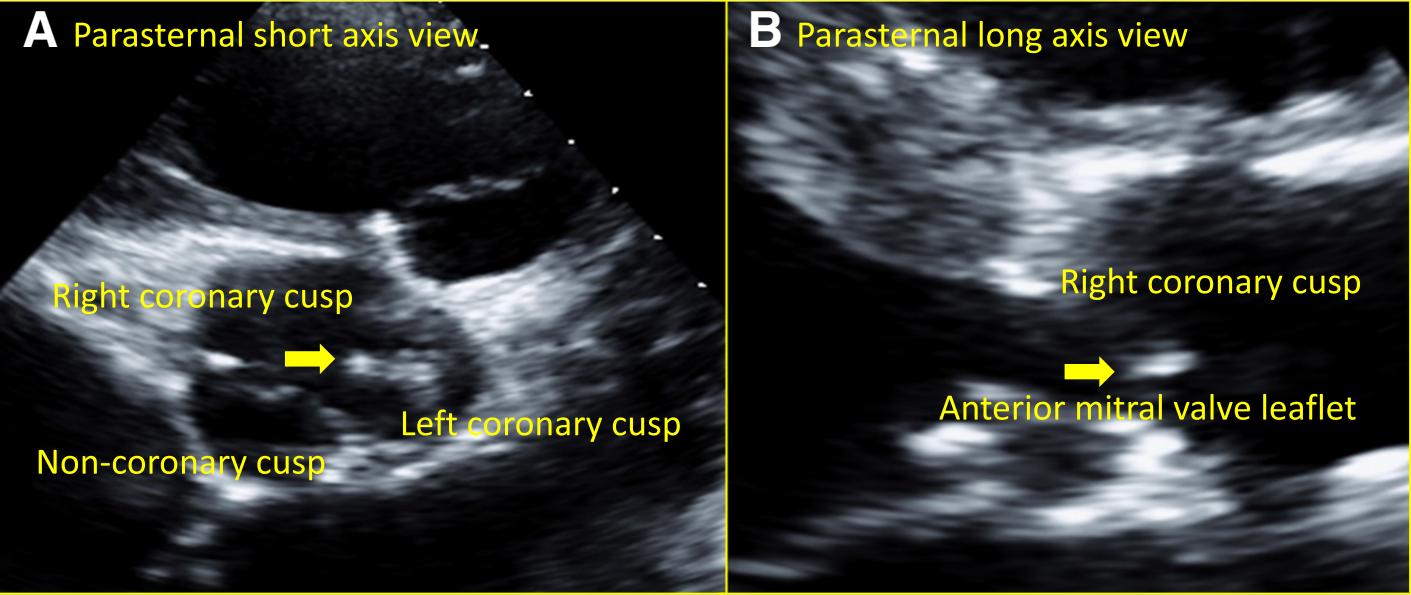
Transthoracic echocardiogram showed two vegetations on the aortic and mitral valves. Legend: Vegetations on the aortic valve (panel **a**) and the mitral valve (panel **b**) were pointed by arrows

**Fig. 2:**
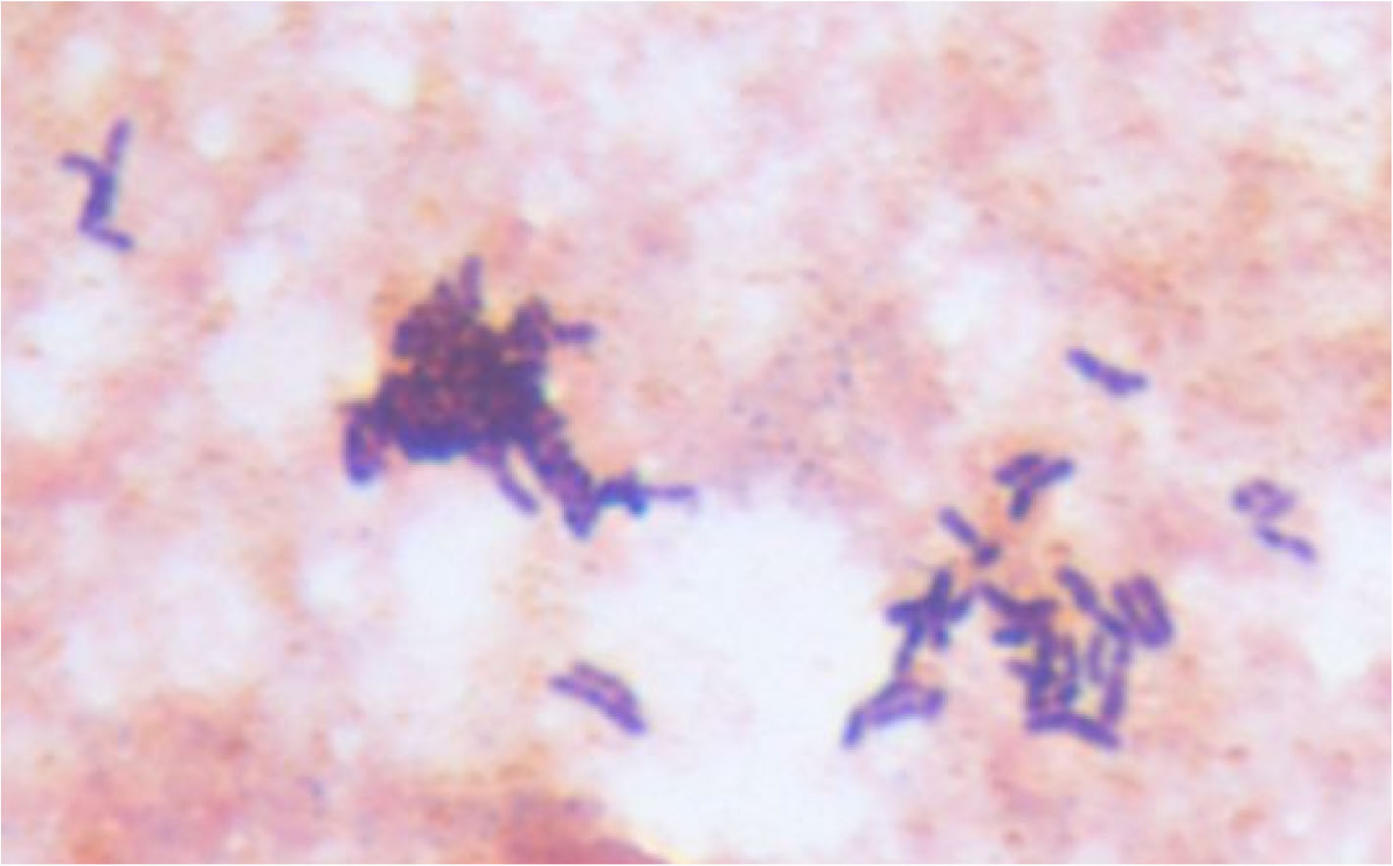
Gram stain morphology of *A. neuii* bacteria. Legend: *A. neuii* bacteria were shown as small, Gram- positive rods. They are non-filamentous and do not produce sulfur granules seen commonly with other *Actinomyces* species

The patient was initially treated empirically for pneumonia and worked up for stroke. However, the treatment plan was quickly modified when a transthoracic echocardiogram on day two of admission revealed two echogenic structures consistent with vegetations: 0.4 × 0.4 cm on the anterior leaflet of the mitral valve, and the other 0.7 × 1.8 cm attached to left coronary cusp of the aortic valve (Fig. 1). There was also thickening of the aortic root suggestive of abscess formation. Two sets of blood culture grew Gram-positive rods after 37.5 h incubating in anaerobic bottles (Fig. 2), and after 86 h in aerobic bottles. The organism was identified as *A. neuii* by MALDI-TOF MS on day five of admission. Serial brain MRI scans revealed multiple bilateral infarcts on day two with increased number of infarcts and a small focus of hemorrhage on day five. The patient was diagnosed with infective endocarditis by *A. neuii* complicated by aortic root abscess and presumed cerebral septic emboli.

### Treatment and outcome

The patient was initially treated with vancomyin and piperacillin/tazobactam until *A. neuii* was identified. Subsequently, he was treated with ampicillin and gentamicin for two days, followed by ampicillin for the rest of his hospitalization. The choice of ampicillin was based on a large series that studied susceptibility to antibiotics of *Actinomyces* species [3], and a previously successfully treated *A. neuii* endocarditis case [4]. Antibiotic susceptibility was not tested for our patient because he responded to the treatment well, and repeat blood cultures were all negative. A CT angiography of the brain and neck on day six ruled out mycotic aneurysm. It was concluded that the risk of further septic embolization outweighed the risk of intracranial hemorrhage, and the patient underwent aortic valve replacement, debridement of aortic root subannular abscess, mitral valve repair, and repair of a fistula between the aorta and left atrium on hospital day fourteen. A 2.5 × 0.6 cm vegetation on the aortic valve and a vegetation on the mitral chordae tendineae were removed. There was no microscopic evidence of bacterial elements on the aortic valve based on histopathology with Gram stain, and culture did not grow any organisms. The patient’s post-operative course was complicated by shock requiring intraaortic balloon pump, and a cardiac arrest from ventricular fibrillation 10 days after surgery. He recovered without further neurological deterioration, and was discharged to a nursing facility two months after heart surgery. He received 12 weeks of IV ampicillin followed by 11 months of oral doxycycline.

One year after the diagnosis of *A. neuii* endocarditis, while on chronic doxycycline, the patient had a fever and a bacteremia with coagulase negative *Staphylococcus* and group B *Streptococcus*. The bacteremia was sterilized after the initiation of antibiotic therapy and there was no growth from subsequent blood cultures. Transthoracic echocardiogram showed a small, mobile echogenic density on the non-coronary cusp of the bioprosthetic aortic valve. The patient refused to undergo transesophageal echocardiogram to further evaluate the prosthetic valve, so he was treated empirically for possible prosthetic valve endocarditis. The patient was cured from infection after two weeks of IV vancomycin and gentamicin, followed by four weeks of IV vancomycin. He had been taking oral doxycycline in addition to his IV antibiotics.

The patient eventually died of a sudden cardiac arrest after hemodialysis. This was 15 months after the diagnosis of *A. neuii* infective endocarditis, and four weeks after discontinuation of oral doxycycline. The family declined autopsy.

### Literature review

Primary infective endocarditis caused by *Actinomyces* spp. is rare. After PubMed (search term ((actinomyces spp) OR actinomyces) AND ((infective endocarditis) OR endocarditis)) and additional bibliographical search, we found 26 human cases dating back to 1939 (Table 1), after excluding four reports, two with bacteria that have been subsequently reclassified to different genera [29, 30], one report with possible direct extension of pulmonary actinomycosis to the endocardium [31], and one with primary IUD-associated actinomyosis and secondary endocarditis [32]. Cases were reported at all ages (6–87 years old). Two thirds of patients were men. The most commonly identified species were *A. israelii* (19%) and *A. viscosus* (15%). Twenty-two cases involved left-sided valves (mitral 9; aortic 5; prosthetic aortic 3; both mitral and aortic 4; undetermined 1). Risk factors included valvular disease (41%), poor dental hygiene or dental procedure (36%), and prosthesis (14%). All four right-sided cases were associated with intravenous drug use [17, 19, 22, 25].

**Table 1:** Review of 26 previously published primary infective endocarditis cases caused by *Actinomyces* species

Author	Year	Sex	Age	Duration of symptoms	Valve(s)	Predisposing factor(s)	Organism	Diagnosis	Therapy	Complication	Outcome
Uhr [5]	1939	M	24	1 month	MV, AV	None	Actinomyces bovis	Autopsy	Sodium iodide	Septic emboli (lungs, small intestine, kidneys)	Died
Beamer [6]	1945	M	55	9 months	MV, AV	Dental caries	Actinomyces graminis	Autopsy	None	Septic emboli (spleen, kidneys, brain)	Died
Mac Neal [7]	1946	M	39	6 weeks	MV	Heart murmur	Actinomyces septicus	Clinical	PCN	Septic emboli (skin, mucosa, brain)	Survived
Wedding [8]	1947	M	37	NA	MV	Rheumatic heart	Actinomyces spp.	Clinical	Sulfathiazole	Septic emboli (spleen, ileum, kidneys, mucosa, brain)	Died
Wedding [8]	1947	F	71	NA	AV	Rheumatic heart	Actinomyces spp.	Autopsy	None	NA	Died
Walters [9]	1962	F	43	2 months	MV	Rheumatic heart. Dental caries	Actinomyces bovis	Clinical	PCN	Septic embolic (mesentery, skin)	Survived
Dutton [10]	1968	M	6	NA	MV	Rheumatic heart	Actinomyces israelii	Autopsy	PCN	CHF. Arrhythmia	Died
Gutschik [11]	1976	M	70	5 months	Left side	Dental abscess	Actinomyces viscosus	Clinical	PCN	Aphasia. Diplopia. CHF	Survived
Lam [12]	1993	M	65	4 weeks	MV, AV	Rheumatic heart. Endocarditis history	Actinomyces israelii	Clinical	PCN	None	Survived
Moffatt [13]	1996	M	48	> 2 weeks	AV	None	Actinomyces meyeri	Clinical and surgical	PCN. Surgery	CHF. Aortic root abscess	Survived
Hamed [14]	1998	M	81	2–3 weeks	AV	Poor dental hygiene	Actinomyces viscosus	Clinical	PCN allergy. Ceftizoxime and ceftriaxone	None	Survived
Huang [15]	1998	F	55	NA	MV	None	Actinomyces meyeri	Clinical	Ampicillin/sulbactam	None	Survived
Mardis [16]	2001	M	38	2 weeks	MV	None	Actinomyces viscosus	Clinical	Vancomycin/gentamicin/cefotaxime ➔ PCN	Cutaneous emboli	Survived
Westling [17]	2002	F	40	2 weeks	TV	IVDU. Endocarditis history	Actinomyces funkei	Clinical	Cefuroxime ➔ cefuroxime/ clindamycin/rifampicin ➔ ceftriaxone ➔ clindamycin	Pulmonary emboli	Survived
Julian [18]	2005	F	43	2 weeks	AV	Bicuspid AV. Dental cleaning	Actinomyces viscosus	Clinical	Ampicillin/azithromycin ➔ vancomycin/gentamicin/ceftriaxone ➔ surgery ➔ vancomycin/ceftriaxone	CHF	Survived
Oh [19]	2005	M	33	2 months	TV	IVDU. Dental procedure	Actinomyces odontolytica	Clinical	PCN/metronidazole	Pulmonary emboli	Survived
Cohen [4]	2007	M	68	3 weeks	AV	Bicuspid AV. Dental procedure	Actinomyces neuii	Clinical	Ampicillin/gentamicin/ceftriaxone ➔ ampicillin ➔ ceftriaxone ➔ doxycycline	Aortic root abscess	Survived
Oddo [20]	2007	M	34	NA	MV	Rheumatic heart. Endocarditis history	Actinomyces spp.	Autopsy	NA	Multi-organ failure	Died
Jitmuang [21]	2008	M	46	1 month	MV	None	Actinomyces georgiae	Clinical	PCN ➔ceftriaxone ➔ ampicillin ➔ amoxicillin	CHF	Survived
Kennedy [22]	2008	F	27	2 days	EV	IVDU. Endocarditis history	Actinomyces israelii	Clinical	Surgery. Unclear antibiotic	Pulmonary emboli	Unclear
Adalja [23]	2010	M	87	2 months	MV	Dental cleaning	Actinomyces israelii	Clinical	PCN	None	Survived
Grundmann [24]	2010	M	66	2 months	PAV	Prosthetics	Actinomyces neuii	Clinical	PCN/meropenem/erythromycin ➔ PCN ➔ amoxicillin. No surgery	Aortic root abscess	Survived
Mehrzad [25]	2013	M	49	NA	TV	IVDU	Actinomyces spp.	Clinical	Vancomycin/ceftriaxone,/ciprofloxacin/metronidazole	Septic emboli (lungs, skin, spleen). Glomerulonephritis.	Survived
Morgan [26]	2014	M	67	6 weeks	PAV	Prosthetics. Dental cleaning	Actinomyces naeslundii	Clinical	Ceftriaxone	Arrhythmia. Septic shock	Died
Cortes [27]	2015	F	51	2 months	PAV	Prosthetics. Dental implant	Actinomyces naeslundii	Clinical	Ceftriaxone ➔ ertapenem ➔ amoxicillin	Roth spots.	Survived
Toom [28]	2018	F	55	8 months	MV, AV	HOCM with LVOT obstruction	Actinomyces israelii	Clinical	PCN	Severe hemolytic anemia	Survived

*M* male, *F* female, *MV* mitral valve, *AV* aortic valve, *TV* tricuspid valve, *EV* Eustachian valve, an embryologic remnant of the valve of the inferior vena cava, *PAV* prosthetic aortic valve, *NA* not available, *PCN* penicillin, *CHF* congestive heart failure, *IVDU* intravenous drug use, *HOCM* hypertrophic obstructive cardiomyopathy, *LVOT* left ventricular outflow tract

Most left-sided endocarditis patients had indolent courses. This did not seem to vary over time. However, the mortality and complications have improved significantly over time. Five of eight patients reported before 1990 died and five had embolic events (brain, spleen, kidneys, small bowel and skin), whereas only two of 14 cases reported after 1990 died, and only one had emboli to skin. Despite temporal courses of a subacute endocarditis, where stigmata of endocarditis are more common, only one report described Roth’s spots [27]. It is unclear whether this was related to virulence factors from *Actinomyces* spp., or simply the rarity of these complications [33]. Right-sided endocarditis cases had more acute and fulminant courses, and were all complicated by septic emboli to the lungs. Two (50%) of them had polymicrobial endocarditis [19, 25], which might have contributed to more complicated clinical courses. All four right-sides cases survived and all were reported after the year of 2000. Irrespective of the side of endocarditis, most patients were treated with a prolonged course of penicillin or β-lactam antibiotics. Four cases had surgery (three aortic valves [4, 13, 18] and one Eustachian valve [22], an embryologic remnant of the valve of the inferior vena cava).

Two cases of infective endocarditis by *A. neuii* were previously reported [4, 24]. Both were in older men with preexisting aortic valvular anomalies (one had a bicuspid valve and the other a prosthetic valve). Both presented with subacute endocarditis, large aortic vegetations (2 cm) and root abscesses. The patient with a native valve underwent surgery [4]. Both patients were cured from the infection. One was initially treated with ampicillin, then ceftriaxone due to interstitial nephritis, and finally doxycycline for 9 months [4]. The other was treated with penicillin, followed by amoxicillin for 12 months [24].

## Discussion and conclusion

Infections caused by *Actinomyces* species, including classic actinomycosis and a range of other infections, usually have indolent courses and favorable outcomes [2]. This pattern was also supported by our review of endocarditis patients. *Actinomyces* species are also very susceptible to antibiotics, except for metronidazole [3, 34]. Such susceptibility to antibiotics, along with advances in diagnosis and management of infective endocarditis, likely contributed to the temporal drop of mortality and systemic complication rates observed from our literature review. Therefore, it was unexpected for our patient to present with an acute course and severe complications. Little is known about the virulence properties of *Actinomyces* spp. [2], but we hypothesize that the valvular damage from previous endocarditis and relative immune deficiency from his end stage renal disease and monoclonal gammopathy may have weakened our patient’s host defense mechanism, and consequently led to a more fulminant course from a pathogen of lower virulence.

*A. neuii* was classified to the genus of *Actinomyces* in 1994. It is a small, non-filamentous rod that does not produce sulfur granules commonly seen in other *Actinomyces* species. Unlike most *Actinomyces* spp. that are anaerobic or at best aerotolerant organisms, *A. neuii* grows in both anaerobically and aerobically incubated samples [35]. It was the third most common diphtheroid and the most common *Actinomyces* species isolated from a tertiary center [36]. It has been reported in infected atheromas [37], abscesses [37], infected foreign bodies [2], urine [36] and endophthalmitis [38, 39]. There have been only a few case reports of classic actinomycosis caused by *A. neuii*. Notably they were all related to breast infections [40–44]. The infection caused by it is thought to be endogenous [35]. The affinity of *A. neuii* to atheromas was only reported in one of the earliest reports, and how infections in atheromas were determined is unclear [37]. However, with such propensity to endovascular infection, it is possible that frequent cannulation for hemodialysis might have contributed to our patient’s infection by *A. neuii*. The outcomes from infections by *A. neuii* are favorable [45]. Given the paucity of cases, our antibiotic selection was based on a previously successfully treated *A. neuii* endocarditis case [4]. The evaluation of neurological complications, and the timing of surgery were challenging, but our management was in line with the latest surgical guideline [46]. The patient’s subsequent possible prosthetic valve endocarditis and eventual death likely reflected his overall poor prognosis, rather than recurrent *A. neuii* endocarditis.

Gram-positive rods, “diphtheroid” or “coryneform”, are often disregarded as contaminants from skin or mucosal surfaces, but 20% of diphtheroid isolates were found to cause clinically significant infections in a large study [36]. *Actinomyces* spp. are among these Gram-positive rods, and their identification in clinical microbiology laboratories can be challenging [2, 47]. As such, delayed diagnoses are common [13, 18, 23], and it is thought endocarditis by *Actinomyces* spp. is underestimated and *Actinomyces* spp. are likely a cause of culture negative endocarditis. Advances in laboratory methods, primarily MALDI-TOF MS, are correctly and increasingly identifying *Actinomyces* spp. from clinical samples. Clinicians should carefully evaluate the relevance of an *Actinomyces* spp. isolate before disregarding it, especially in a vulnerable patient like ours, and in a species that is associated with endovascular infection like *A. neuii*.

To conclude, we reported a successfully treated acute infective endocarditis case with severe complications by *A. neuii*, a rare but increasingly clinically relevant *Actinomyces* species associated with endovascular infection. Our review showed *Actinomyces* spp. infective endocarditis is usually indolent and responds favorably to treatment. Clinicians should carefully evaluate the relevance of *Actinomyces* spp. in infections to avoid delayed or missed diagnoses.

## Data Availability

Not applicable. No datasets were generated for this study.

## Abbreviations

CT: Computed tomography
GI: Gastrointestinal
IUD: Intrauterine device
IV: Intravenous
MALDI-TOF MS: Matrix-assisted laser desorption ionization time-of-flight mass spectrometry
MRI: Magnetic resonance imaging
